# Ozone Exposure Induces Prediabetic Symptoms Through Hepatic Glycogen Metabolism and Insulin Resistance

**DOI:** 10.3390/toxics13080652

**Published:** 2025-07-31

**Authors:** Yuchai Tian, Xiaoyun Wu, Zhihua Gong, Xiaomin Liang, Huizhen Zhu, Jiyue Zhang, Yangcheng Hu, Bin Li, Pengchong Xu, Kaiyue Guo, Huifeng Yue

**Affiliations:** 1 Shanxi Key Laboratory of Coal-Based Emerging Pollutant Identification and Risk Control, Research Center of Environment and Health, College of Environment and Resource, Shanxi University, Taiyuan 030006, China; 202313905004@email.sxu.edu.cn (Y.T.); 202023902017@email.sxu.edu.cn (X.W.); gongzhihua@sxbqeh.com.cn (Z.G.); 202413905003@email.sxu.edu.cn (X.L.); 202223905013@email.sxu.edu.cn (H.Z.); 202323905009@email.sxu.edu.cn (J.Z.); 202323905004@email.sxu.edu.cn (Y.H.); 202423902006@email.sxu.edu.cn (B.L.); 202123905010@email.sxu.edu.cn (P.X.); 2 Department of Clinical Laboratory, Shanxi Bethune Hospital, Shanxi Academy of Medical Sciences, Third Hospital of Shanxi Medical University, Tongji Shanxi Hospital, Taiyuan 030032, China; 3 Shanxi Province Key Laboratory of Oral Diseases Prevention and New Materials, Shanxi Medical University School and Hospital of Stomatology, Taiyuan 030001, China; guokaiyue19942024@163.com

**Keywords:** ozone exposure, glucose tolerance, liver injuries, prediabetes

## Abstract

(1) Background: Epidemiological studies link ozone (O_3_) exposure to diabetes risk, but mechanisms and early biomarkers remain unclear. (2) Methods: Female mice exposed to 0.5/1.0 ppm O_3_ were assessed for glucose tolerance and HOMA (homeostasis model assessment) index. Genes related to impaired glucose tolerance and insulin resistance were screened through the Comparative Toxicogenomics Database (CTD), and verified using quantitative real-time PCR. In addition, liver histopathological observations and the determination of basic biochemical indicators were conducted, and targeted metabolomics analysis was performed on the liver to verify glycogen levels and gene expression. In vitro validation was conducted with HepG2 and Min6 cell lines. (3) Results: Fasting blood glucose and insulin resistance were elevated following O_3_ exposure. Given that the liver plays a critical role in glucose metabolism, we further investigated hepatocyte apoptosis and alterations in glycogen metabolism, including reduced glycogen levels and genetic dysregulation. Metabolomics analysis revealed abnormalities in fructose metabolism and glycogen synthesis in the livers of the O_3_-exposed group. In vitro studies demonstrated that oxidative stress enhances both liver cell apoptosis and insulin resistance in pancreatic islet β cells. (4) Conclusions: O_3_ triggers prediabetes symptoms via hepatic metabolic dysfunction and hepatocyte apoptosis. The identified metabolites and genes offer potential as early biomarkers and therapeutic targets.

## 1. Introduction

Since the latter half of the 20th century, air pollution has been on the rise due to gradually accelerating industrialization processes [1]. As a result of the strong measures implemented by the Chinese government, pollution levels of some pollutants such as PM_2.5_ and PM_10_ have significantly decreased while environmental ozone (O_3_) levels are still showing an overall upward trend [2]. Besides natural emissions, most of the ground-level O_3_ originates from the photochemical reactions among human activity-related pollutants such as volatile organic compounds and nitrogen oxides [3]. In 2019, the WHO’s warm season O_3_ standard was exceeded by 96% of the 12,946 cities surveyed, with an average annual O_3_ exposure of 51 ppb [4]. According to the World Meteorological Organization Air Quality and Climate Bulletin (2022), average global temperatures are anticipated to rise by 3 °C above pre-industrial levels, causing sustained elevated O_3_ levels, mostly in Asia. The study by Shen et al. showed that the maximum daily 8 h average O_3_ concentration in the warm season in 74 major cities in China is increasing at an annual rate of 3% [5].

In 2019, the “global burden of disease” estimated that around 400,000 deaths could be attributed to atmospheric O_3_ pollution [6]. Causes of death include not only exacerbations of asthma, chronic obstructive pulmonary disease, and inflammation of the lungs [7], but also a significant proportion of cardiovascular diseases, such as elevated blood pressure caused by vasoconstriction [8]. It has been reported that disorders of glucose metabolism are the underlying cause of prediabetes. Prediabetes includes both impaired fasting plasma glucose and abnormal glucose tolerance with persistent insulin resistance [9]. O_3_ exposure also impairs insulin signaling by sequentially triggering oxidative stress, endoplasmic reticulum stress, and activation of the JNK signaling pathway [10]. Several follow-up studies over 10 years have shown that O_3_ exposure not only increases the risk of developing type 2 diabetes in humans, but also accelerates the progression of diabetes [11,12].

Epidemiologic studies have confirmed the correlation between O_3_ exposure and diabetes, but the specific mechanisms of action need to be further explored. In this study, we measured whether chronic exposure to O_3_ leads to abnormal glucose tolerance in adult female mice, and explored the effects of damage to the liver, as a hub that regulates blood glucose, on this process, and on glycolipid metabolic processes, as well as exploring potential biomarkers and mechanisms.

## 2. Materials and Methods

### 2.1. Animal Experiments

BALB/c female mice (7–8 weeks old) were obtained from Beijing Vital River Laboratory Animal Technology Co. (Beijing, China). After a week of acclimatization, the animals were housed under standard laboratory conditions with ad libitum access to food (Beijing Vital River Laboratory Animal Technology Co., Ltd.) and water. The mice were randomly allocated into three groups: control, 0.5 ppm, and 1.0 ppm (n = 9) and exposed to the corresponding concentration of O_3_ for 4 h daily, continuously for 28 days. The basis for the exposure concentrations is placed in Appendix A. All care, treatment, and experimental protocols were approved by the Scientific Research Committee of Shanxi University (SXULL2022077, Taiyuan, China).

### 2.2. Glucose Tolerance Test

As described by Small et al. [13], the experimental protocol was initiated at 22:00, wherein mice were subjected to a 12 h fasting period. Their FPG levels were then analyzed by acquiring blood samples from the tail vein. After that, a 2 g/kg dose of glucose delivered in a 40% glucose solution was then administered intraperitoneally, and measurements of plasma glucose via a hand-held glucometer were obtained at 0.5 h, 1.0 h, 1.5 h, and 2.0 h post-injection. The resulting data are presented as time–glucose concentration curves, which enables the evaluation of the glucose tolerance of the mice via area under the curve (AUC) values.

### 2.3. Enzyme-Linked Immunosorbent Assay (ELISA) for Insulin

Blood samples were collected from the mice at the end of the 28-day exposure and allowed to clot naturally for 3–4 h. Afterward, the samples were subjected to centrifugation for 15 min at a rate of 3500 revolutions per minute. Careful collection of the supernatant followed to ensure the retrieval of serum, which was stored at a temperature of −80 °C. The serum insulin content was then assayed according to the manufacturer’s instructions (JL11459; Jianglai Biology, Shanghai, China).

### 2.4. Calculation of the Homeostatic Model Assessment (HOMA) Index

HOMA-IR is the indicator used to evaluate an individual’s level of insulin resistance [14]. It is calculated using the following formula:

[fasting insulin (FINS) (μU/mL) × fasting plasma glucose (FPG) (mmol/L)]/22.5

HOMA-IS is the indicator used to evaluate an individual’s level of insulin sensitivity. It is the inverse of HOMA-IR.

HOMA-β is the indicator used to evaluate an individual’s level of islet β-cell function. It is calculated using the following formula:

[20 × FINS (μU/mL)]/[FPG (mmol/L) − 3.5]

### 2.5. Hematoxylin-Eosin (H&E) Staining

The liver samples were washed with a saline solution and fixed in 10% formalin for an overnight period. Subsequently, the tissue was embedded in paraffin, sectioned to a thickness of 5–6 μm, and processed with H&E staining [15]. The prepared liver sections were examined under a microscope with 100× magnification.

### 2.6. Measurement of Liver Metabolism Indicators

Serum levels of lactate dehydrogenase, urea, cholesterol, triglycerides, and both low-density and high-density lipoprotein were measured in mice using an automated biochemical analyzer (AU5800, Beckman Coulter Inc., Brea, CA, USA) according to the manufacturer’s guidelines.

### 2.7. Liver Glycogen Quantification

In this study, a Micro Glucogen Content Assay Kit was utilized to quantify the amount of glycogen present in liver tissue samples. The experimental protocol followed the guidelines provided by the manufacturer of the kit (BC0345, Beijing Solarbio Science & Technology Co., Ltd., Beijing, China). This method utilized a strong alkaline extract to extract glycogen, and anthrone color developer under strong acidic conditions was used to determine glycogen content.

### 2.8. Immunohistochemical (IHC) Staining

IHC staining was conducted following established protocols [16]. Briefly, paraffin sections were subjected to dewaxing with xylene and gradient ethanol, followed by sequential steps of antigen retrieval, membrane permeabilization, endogenous peroxidase blocking, and goat serum blocking. Bax primary antibody (1:200, rabbit anti-Bax antibody, Bioss, Beijing, China, bs-1027R) was incubated overnight at 4 °C. The staining was visualized with diaminobenzidine (DAB) (Beijing Zhongshan Jinqiao Biotechnology Co., Ltd., Beijing, China) followed by hematoxylin counterstaining. The sections were inspected under an optical microscope and photographed. For each sample, five random microscopic fields were designated for analysis. The positive signal regions were screened via Image J software, and the percentage of the positive area within the entire section area (Area%) was calculated for statistical analysis. During the statistical process, a blinded approach was employed. Specifically, all the sections from all groups were randomly shuffled, and then the area of the positive regions was measured using Image J software.

### 2.9. Central Carbon Metabolite Analysis

The targeting assay for central carbon metabolites in the livers of the control and 1.0 ppm groups was performed by Majorbio (Shanghai, China). As described by Willacey et al. [17], the standard solutions of 49 central carbon metabolites were configured, and the standard curves of different concentrations were made. For each group of 5 samples, 10 mg of liver tissue was accurately weighed, and ground using a cryomill for 6 min (−10 °C, 50 Hz), then the extract was added to the extract solution for metabolite extraction, and the supernatant was taken as the sample to be tested, and then the standard solution and the sample to be tested were subjected to LC-MS under the same conditions, and the concentrations of the target substances in the test samples were calculated according to the standard curves, and then converted into the actual content of the target substances in the samples. The data were analyzed by Orthogonal Partial Least Squares Discriminant Analysis (OPLS-DA) using the online website Wekemo Bioincloud (https://www.bioincloud.tech/, accessed on 13 December 2023). The specific operation process and parameters are as follows: After opening the website, select “Cloud Tools”, enter the data in the sample format, and set the number of replacements to 500. Metabolites satisfying both variable important in projection (VIP) ≥1 and fold change ≤0.67 or ≥1.5 were considered as differential metabolites. Enrichment analyses of metabolites were performed using MetaboAnalyst 5.0.

### 2.10. Diseases Search

As described by Davis et al. [18], the Comparative Toxicogenomics Database (CTD) (http://ctdbase.org/) was employed to examine diseases associated with O_3_ exposure (latest search time: 4 October 2023). Specifically, we searched the chemical database with the keyword “ozone” and clicked on “Diseases” to display O_3_-related diseases and the genes associated with the disease.

### 2.11. Quantitative Real-Time PCR (qPCR)

Briefly, we first weighed mouse liver tissue on an ice box. Total RNA was extracted, gDNA was removed to allow reverse transcription to cDNA, and then it was amplified by qPCR. The total amount of product after each PCR cycle was measured with fluorescent chemicals, using the housekeeping gene *glyceraldehyde-3-phosphate dehydrogenase* (*Gapdh*) as internal reference. The detailed protocols have been expatiated in Text A2 and the utilized primer sequences have been documented in Table A1. The methodology has been cited in the published research by [19].

### 2.12. Cell Culture

HepG2 and Min6 cells were routinely cultured using DMEM medium containing 10% fetal bovine serum and 100 U/mL penicillin/streptomycin solution. The culture conditions were set at 5% CO_2_, 95% humidity, and 37 °C constant temperature. The cell culture protocol was referenced by Yu et al. [20].

### 2.13. Measurement of Cell Apoptosis

The experimental method of apoptosis flow cytometry has been described in our previous articles [21]. HepG2 cells (1.5 × 10^5^/mL) were seeded in 35 mm culture dishes and cultured for 24 h. Fresh medium was added to the control group, and oxidative stress was induced in the treatment group by the addition of medium containing H_2_O_2_ (200 μmol/L). Cells were collected after 12 h and washed twice with PBS. Subsequently, they were incubated with Annexin V-FITC and PI probes in the dark for 15 min. Detection was carried out using a flow cytometer (for Annexin V-FITC: excitation at 488 nm, emission at 525 nm, BL1; for PI: excitation at 488 nm, emission at 630 nm, YL1). After fluorescence compensation calibration, 10,000 valid signals were recorded for each sample.

### 2.14. Glucose Consumption Experiment

Cells were treated as described above, and after 12 h of induced oxidative stress, they were stimulated by the addition of insulin (100 nM) for 30 min. To assess the effect of oxidative stress on glucose uptake in HepG2 cells, the glucose content of the culture medium was assayed using the Glucose Content Assay Kit (BC2505, Beijing Solarbio Science & Technology Co., Ltd.), according to the manufacturer’s instructions. The experimental method was referred to by Liu et al. [22].

### 2.15. Glucose Tolerance Test of Min6 Cells

Min6 cells were seeded in 24-well culture plates (5 × 10^5^ cells/well) and cultured in DMEM medium containing 15% fetal bovine serum at 37 °C for 48 h. Then, serum-free medium without or with H_2_O_2_ was replaced in the control and exposure groups, respectively. After 12 h, the cells were washed with PBS, and 25 mM glucose-containing medium was added to each well and the cells were incubated at 37 °C for 1 h. The supernatant was collected, and then the medium was replaced with 75 mM glucose-containing medium and incubated at 37 °C for 1 h. The supernatant was collected again. The insulin content in the supernatant under basal and high glucose conditions was determined, and the stimulation index (SI) was calculated [23]:

SI = (the insulin content stimulated by high glucose solution)/(the insulin content stimulated by low glucose solution)

### 2.16. Statistical Analysis

The statistical outcomes were presented as mean ± standard deviation (SD). Due to the small sample size, we employed non-parametric tests. The experimental results among the three groups were examined using the Kruskal–Wallis H test, and Mann–Whitney U test was used for comparison between the two groups in SPSS 26.0. Statistical significance was determined when *p* < 0.05. Graphs were generated with GraphPad Prism 8.0.1 and online website bioinformatics (https://www.bioinformatics.com.cn/). The IHC Toolbox plugins in Image J (1.51 j8) was utilized to calculate the positive area for immunohistochemical staining sections.

## 3. Results

### 3.1. Exposure to O_3_ Leads to Impaired Glucose Tolerance and Insulin Resistance

**Figure 1:**
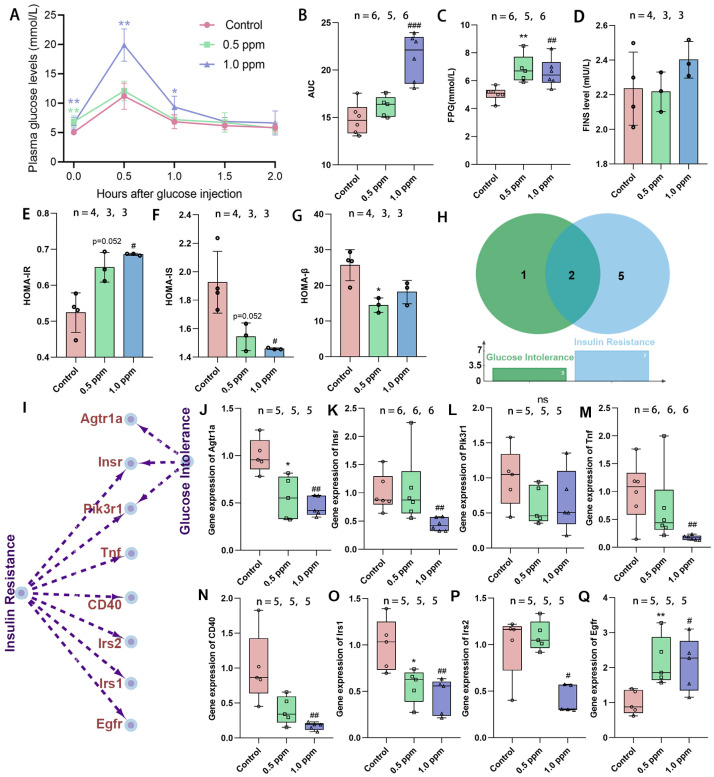
The effects of O_3_ exposure on glucose tolerance. (**A**) Time–glucose concentration curve after tail vein injection of glucose. (**B**) Time–glucose concentration area under the curve. (**C**) Fasting plasma glucose concentration. (**D**) Fasting insulin levels. (**E**) Insulin resistance index. (**F**) Insulin sensitivity index. (**G**) Islet β cell function index. (**H**) The number and (**I**) specific corresponding relationships of genes involved in O_3_-related glucose intolerance and insulin resistance retrieved from the CTD database. (**J**–**Q**) Relative expression of the genes *Agtr1a*, *Insr*, *Pik3r1*, *Tnf*, *CD40*, *Irs1*, *Irs2*, and *Egfr*. The value is the mean ± SD; 0.5 ppm: * *p* < 0.05, ** *p* < 0.01. 1.0 ppm: # *p* < 0.05, ## *p* < 0.01, ### *p* < 0.001, compared with the control group. The number of samples (n) in each group is marked in the figure.

Initially, the study assessed the effects of exposure to O_3_ on glucose tolerance in adult female mice by monitoring plasma glucose levels in both a fasting state and following intraperitoneal glucose administration (0.5, 1.0, 1.5, and 2.0 h) and computing the AUG concentration curves (Figure 1A). It was found that the AUC in the 1.0 ppm group increased significantly, while the 0.5 ppm group showed an upward trend (Figure 1B). Statistically significant elevations in FPG levels were observed in the 0.5 ppm and 1.0 ppm groups when compared to the control group (Figure 1C). Correspondingly, post glucose injection, a significant increase in plasma glucose values was found in the 1.0 ppm group at 0.5 h and 1.0 h in comparison to the control. At 1.5 and 2.0 h after glucose injection, plasma glucose returned to normal levels (Figure 1A). Elevated plasma glucose may result from insufficient insulin secretion, insulin resistance, abnormal glycogenolysis, or gluconeogenesis, among other factors. We further measured fasting insulin levels, yet no significant differences were observed (Figure 1D). Subsequently, the HOMA index was calculated to assess the insulin resistance, insulin sensitivity, and β-cell function. The results indicated that the 0.5 ppm and 1.0 ppm O_3_-exposed groups showed significantly higher insulin resistance (0.5 ppm: 1.24-fold vs. control; 1.0 ppm: 1.31-fold vs. control), along with lower insulin sensitivity and islet β-cell function compared to the control group (Figure 1E–G).

To substantiate our findings, we used the CTD database to trace the O_3_–disease–gene pathway and determine genes associated with insulin resistance and abnormal glucose tolerance (Figure 1H,I). As indicated, gene expressions of *Agtr1a* and *Irs1* were significantly lower in both the 0.5 ppm and 1 ppm O_3_ groups compared to the control group, whereas the *Egfr* gene was significantly higher and the *Insr*, *Tnf*, *CD40*, and *Irs2* genes were lower only in the 1.0 ppm group (Figure 1J–Q). Of these, *Insr* encodes the insulin receptor, and *Irs1* and *Irs2* encode insulin receptor substrates, and their reduced expression implies impaired synthesis of the liver’s insulin receptor. This confirms that O_3_ exposure increases the risk of insulin resistance and abnormal glucose tolerance in adult female mice.

### 3.2. The Pathways Through Which O_3_ Exposure Affects Blood Glucose Levels

**Figure 2:**
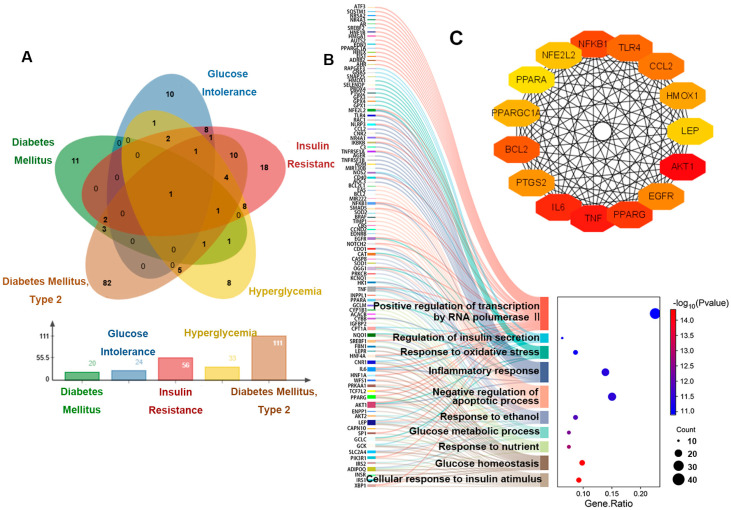
Genetic analysis of hyperglycemia disease associated with O_3_ exposure. (**A**) Venn diagram of genes related to five diseases. (**B**) The relationship between the genes related to five diseases and the enriched pathways. (**C**) Interaction diagram of key genes.

The above results revealed that the mice showed signs of prediabetes, and the diseases listed under our search for “ozone” entry in the CTD database included the following: diabetes mellitus, glucose intolerance, insulin resistance, hyperglycemia and diabetes mellitus, type 2 (Figure 2A). And disease-related genes and disease sequencing are shown in Table A2. Our GO enrichment analysis of these five disease-associated genes showed that the top 10 entries focused on glucose homeostasis, response to oxidative stress, inflammatory response, and apoptotic process (Figure 2B). In addition, we have also screened out the top 15 genes that affect these pathways (Figure 2C).

### 3.3. O_3_ Exposure Induces Intensified Hepatocyte Apoptosis and Lipid Metabolism Disorders

**Figure 3:**
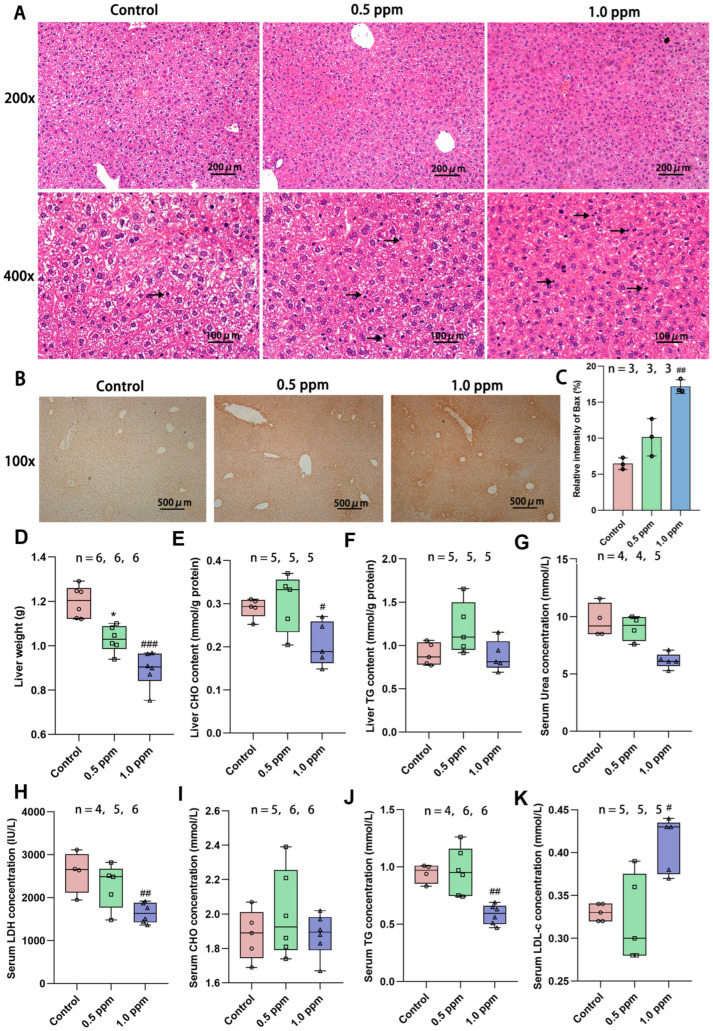
Effects of O_3_ exposure on histopathology and basic indicators of liver health. (**A**) In H&E staining of the liver, compared with the control group, the number of apoptotic cells (marked by black arrow) was significantly increased in the 0.5 ppm and 1.0 ppm groups. (**B**) IHC staining for the pro-apoptotic protein Bax. (**C**) Quantification of IHC-stained positive areas as a percentage of total area (n = 3, five different fields of view were selected for each sample). (**D**) Liver weight. (**E**) Total liver CHO and (**F**) TG content. Serum levels of (**G**) Urea, (**H**) LDH, (**I**) CHO, (**J**) TG, and (**K**) LDL-C. The value is the mean ± SD; 0.5 ppm: * *p* < 0.05, 1.0 ppm: # *p* < 0.05, ## *p* < 0.01, ### *p* < 0.001, compared with the control group. The number of samples (n) in each group is marked in the figure. CHO: cholesterol; TG: triglyceride; LDH: lactate dehydrogenase; LDL-C: low-density lipoprotein cholesterol.

Based on the above enrichment results, we first focused our attention on the liver. The liver is a central regulator of glucose homeostasis, which, when impaired, is thought to be one of the earliest factors contributing to impaired systemic glucose homeostasis [24]. Hepatic insulin resistance is accompanied by elevated rates of hepatic gluconeogenesis and fasting hyperglycemia [25]. In order to further investigate the underlying causes of glucose metabolism disorders, we initially assessed the basic characteristics of the liver. H&E staining was performed on liver sections to determine the histopathological alterations induced by O_3_ exposure. The results revealed that the liver cells in the O_3_-exposed group exhibited nuclear condensation and cytoplasmic shrinkage. The nuclear volume decreased, the color darkened, and some nuclei disappeared, indicating cellular apoptosis (Figure 3A). To further determine whether the apoptosis of liver cells is increased, immunohistochemical staining of the liver sections was performed using the pro-apoptotic protein Bax (Figure 3B), and the positive areas were quantified. The results demonstrated a significant increase in apoptotic cells (control: 6.47%; 1.0 ppm: 17.17%, *p* = 0.007 vs. control) due to O_3_ exposure (Figure 3C). Correspondingly, we found that the basic indicators of the liver were also affected, such as the liver weight (Figure 3D). O_3_ exposure led to a significant reduction in liver weight, particularly in the 1.0 ppm group compared to the control group. Additionally, several blood indicators related to liver metabolism, including triglycerides (TG) and lactate dehydrogenase (LDH) were significantly decreased, while low-density lipoprotein cholesterol (LDL-C) was significantly increased. Cholesterol (CHO) content in the liver decreased in the 1.0 ppm group (Figure 3E–K).

### 3.4. O_3_ Exposure Reduces Liver Glycogen Content and Abnormal Metabolic Products

**Figure 4:**
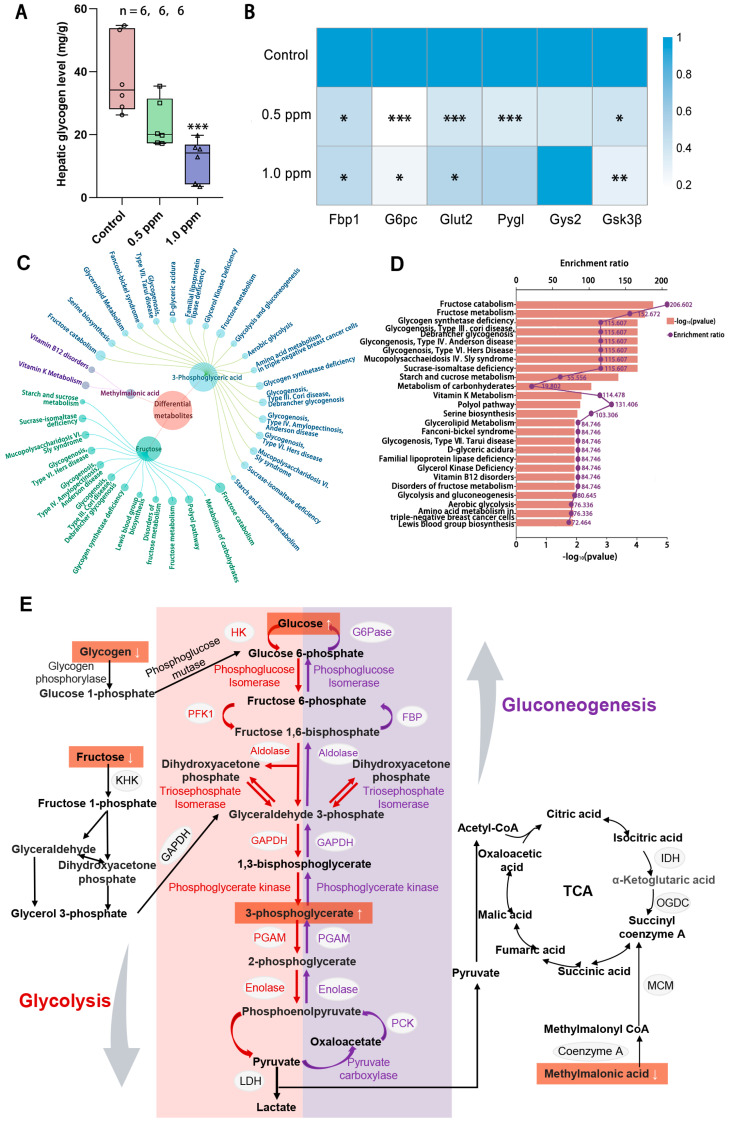
The effects of O_3_ exposure on liver glycogen metabolism. (**A**) Hepatic glycogen level. (**B**) Relative expression of genes *Pygl*, *Fbp1, Gys2, Glut2, Gs3kβ,* and *G6pc*, which are involved in glycogen synthesis and catabolism in the liver. The value is the mean ± SD; * *p* < 0.05, ** *p* < 0.01, *** *p* < 0.001 vs. control group. (**C**) Linkage plot between differential metabolites and the top 25 enriched metabolic sets. (**D**) Schematic representation of the enrichment rate and *p*-value of the metabolite set. (**E**) Metabolite network map of glucose metabolism. The number of samples (n) in each group is marked in the figure.

According to the results for the enrichment of disease-related genes, O_3_ exposure also interferes with glucose homeostasis. The liver serves as a central hub for regulating and maintaining blood glucose balance, as it is the site for glycogen synthesis, decomposition, and gluconeogenesis. Therefore, we quantitatively measured the glycogen content in the liver and found that liver glycogen content showed a decreasing trend with increasing exposure concentrations, with a significant decrease in the 1.0 ppm group (Figure 4A). To further explore the impact of O_3_ on hepatic glucose metabolism, a qPCR analysis of genes involved in glycogen metabolism, namely *Gsk3β*, *Glut2*, *G6pc*, *Fbp1*, *Pygl*, and *Gys2*, was conducted (Figure 4B). The results showed that the expression of hepatic glycogen synthesis genes (glycogen synthase kinase gene *Gsk3β*) and hepatic glycogen catabolism genes (glycogen phosphorylase gene *Pygl*) was significantly decreased after exposure to O_3_, indicating that both hepatic glycogen synthesis and catabolism were impaired. Consequently, a central carbon metabolomics analysis of the liver in the control and 1.0 ppm groups was conducted to assess whether there were any abnormalities in the hepatic glucose metabolism processes. We screened three differential metabolites (Table A3), fructose, 3-phosphoglyceric acid and methylmalonic acid, and enriched them for analysis. The top 25 metabolite sets were significantly enriched, which were mainly associated with fructose catabolism and glycogen synthesis and catabolism, are illustrated in Figure 4C, D. Figure 4E shows the glucose metabolism map and the differential metabolites.

### 3.5. In Vitro Experiments to Verify the Damage Mechanism of O_3_ Exposure

**Figure 5:**
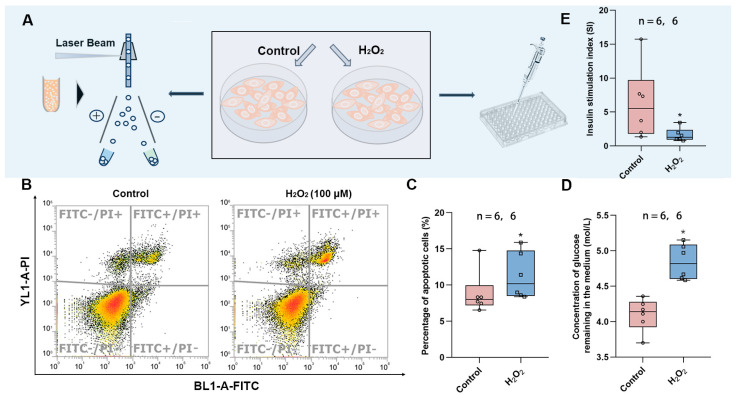
Effects of oxidative stress on HepG2 and Min6 cells. (**A**) Schematic diagram of apoptosis detection and glucose content measurement. (**B**) Representative images of apoptosis measured by flow cytometry. (**C**) Percentage of apoptotic cells in control vs. H_2_O_2_ group. (**D**) The content of glucose remaining in the medium after glucose consumption by insulin. (**E**) Stimulation index of Min6 cells. The value is the mean ± SD; * *p* < 0.05 vs. control group. The number of samples (n) in each group is marked in the figure.

To verify the findings in the animal experiments, we exposed the HepG2 cell line to hydrogen peroxide to induce oxidative stress. This was followed by the detection of apoptosis using flow cytometry and the measurement of glucose uptake in the culture medium to determine whether the cells developed insulin resistance (Figure 5A). It was found that HepG2 cells exposed to H_2_O_2_ showed increased apoptosis (Figure 5B,C), diminished response to insulin, and decreased glucose consumption (Figure 5D), implying that oxidative stress led to insulin resistance. In addition, we also exposed Min6 cells to H_2_O_2_ to induce oxidative stress and found that the stimulation index (SI) was significantly lower in the exposed group, implying that oxidative stress also attenuates the response of islet cells to glucose and decreases insulin secretion in response to high levels of glucose stimulation.

## 4. Discussion

Consistent with our results, published studies have demonstrated that acute exposure to 1.0 ppm O_3_ in juvenile females and adult males results in elevations in plasma glucose levels and glucose intolerance [26,27]. Acute exposure was also noted to cause abnormal glucose tolerance and insulin resistance in mice and humans across all age groups. Moreover, O_3_-induced disruptions in glucose metabolic processes in both juvenile and adult mammals may potentially contribute to diabetic pathogenesis, which needs further examination [28]. Consistent with previous findings of elevated fasting glucose levels in the O_3_-exposed mice, these results suggest that chronic O_3_ exposure induces glucose intolerance, which can be mediated by insulin resistance, impairment of insulin sensitivity, and β-cell dysfunction [29]. Research suggests that O_3_ exposure can interfere with insulin receptor signaling pathways, thereby impacting insulin sensitivity [30].

The *Agtr1a* gene encodes the angiotensin II receptor 1a, which regulates glucose metabolism by modulating the renin–angiotensin–aldosterone system and the sympathetic nervous system [31]. Low expression of *Agtr1a* has been shown to limit abnormal glucose tolerance in previous studies [32,33]. Our hypothesis is that O_3_ exposure activates a negative feedback effect in the body, leading to reduced *Agtr1a* expression and modulation of its own glucose abnormalities. Furthermore, Rong et al. observed insulin resistance and elevated plasma glucose in mice deficient in *Agtr1a* on a high-fat diet [34]. Wolf et al. revealed that *CD40* signaling activation reduces inflammation and metabolic complications in adipose tissue [35], whereas *CD40* deficiency induces impaired insulin secretion and metabolic disorder syndrome [36]. Studies in human populations exposed to PM_2.5_ have shown increased *CD40* expression in circulating cells, leading to systemic inflammation. This phenomenon is amplified in individuals with diabetes who have characteristics associated with insulin resistance or oxidative stress [37]. *Irs1* and *Irs2* encode insulin receptor substrates 1 and 2, which are key molecules in insulin signaling. Studies have shown that the expression of *Irs1* and *Irs2* genes is reduced in diabetic patients [38], and *Irs1* expression is notably reduced in cells cultured under high glucose conditions [39]. The *Insr* gene affects insulin signaling by enzymatically catalyzing phosphorylated substrate proteins (insulin receptor substrates) [40], so its low expression leads to reduced catalytic activity of IRS1 and IRS2. Similar to our findings, the expression of *Insr*, *Irs1*, *Irs2*, and *Aktr1a* was also reduced in the livers of the F3 offspring of mice chronically exposed to advanced glycation end-products (AGEs), along with the development of metabolic impairment [41]. *Tnf* is a tumor necrosis factor and it has been demonstrated that short exposures to PM_2.5_ significantly increase liver inflammatory factors including *Il-6* and *Tnf-α* in rats [42], while the activation of inflammatory factors has been shown to contribute to insulin resistance [43]. The epidermal growth factor receptor (EGFR) is known to be a key factor in glucose metabolism disorders due to its ability to inhibit insulin receptor activity upon activation [44].

Epidemiological studies have shown that O_3_ exposure can lead to the impairment of glucose homeostasis, and systemic inflammation and oxidative stress may be potential pathways through which O_3_ exposure causes damage to glucose homeostasis [45]. One possible mechanism is that chronic exposure to O_3_ results in the release of stress hormones [46] and leads to increased oxidative stress in humans, which is characterized by decreased antioxidant capacity, DNA damage, ROS production, and altered inflammatory factors [47,48], and has been suggested to contribute to the development of insulin resistance. Oxidative stress is the chief culprit for inflammation and apoptosis. Animal studies have also found that the expression of genes involved in apoptosis is abnormal after O_3_ exposure [49]. Our study found that the elevation of blood glucose caused by O_3_ exposure might act through disrupting glucose metabolism and through apoptosis induced by oxidative stress, which prompted us to conduct further verification. Although few studies have demonstrated the relationship between O_3_ exposure and apoptosis in the liver, Güvendi et al. reported increased inflammation and apoptosis in O_3_-exposed groups through liver sections [50]. Excessive apoptosis of hepatocytes can contribute to insufficient liver function and liver disease, such as hepatitis, cirrhosis, and liver cancer. Other air pollutant-related studies have shown that exposure of mice to PM_2.5_ resulted in increased liver inflammatory signaling with macrophage infiltration. There was also an upregulation of hepatic apoptotic effectors, similar to that seen with O_3_ exposure [51]. Moreover, chronic exposure to NO_2_ in the environment increases the chances of developing fatty liver associated with metabolic dysfunction in the population [52]. In healthy liver tissue, balance is achieved through apoptosis; the regulators of apoptosis signaling events in hepatocytes also regulate insulin signaling pathways, and the mediators of insulin resistance in turn affect hepatocyte apoptosis [25]. Therefore, this study found that the liver apoptosis induced by exposure to O_3_ may be the cause of the abnormal elevation of blood glucose levels in mice.

The liver plays a critical role in protein and amino acid metabolism, including the breakdown of amino acids for energy, the secretion of most proteins, and the processing of nitrogenous waste products in the form of urea [53]. The results above are consistent with previous studies on acute and chronic O_3_ exposure, which report overall metabolic disturbances involving glucose, lipid, and amino acid metabolism [49,54]. Notably, LDH is a key enzyme involved in glycolysis, as well as gluconeogenesis, and its reduction largely affects the process of glucose metabolism; the same LDH inhibition was observed in mice with diabetes [55]. However, short-term exposure to both air pollutants, carbon monoxide [56], and PM_2.5_ [57] produced excessive LDH release in the livers of mice, and the opposite result was seen in our O_3_ exposure study. Urea levels in the serum reflect the metabolic and excretory functions of the liver [58]. A population-based analysis by Mao et al. showed a negative correlation between serum urea levels and the prevalence of diabetes in adults [59], and both PM_2.5_ and O_3_ exposure interfere with hepatic urea production [60]. LDL-C is a primary cholesterol carrier in the blood [61], and in our study, O_3_ exposure resulted in elevated LDL-C levels in female mice. This is the same result as that is produced by PM_10_ and NO_2_ exposure [62]. Its elevation causes diabetes mediated by insulin resistance and inflammation [63]. Additionally, TG is a critical energy storage substance, and decreased plasma TG occurs in hyperlipidemic and diabetic mice that are chronically exposed to SO_2_ [64].

Previous studies have theorized a potential relationship between O_3_ exposure and hepatic gluconeogenesis [65], but conclusive evidence has been lacking. The present study provides an empirical basis for this hypothesis by revealing the impacts of O_3_ on both quantitative hepatic glycogen analysis and hepatic glucose metabolism gene expression. Among the examined genes, *Gs3kβ*, a pivotal enzyme regulating the balance between glycogen synthesis and glycogen catabolism, showed reduced expression, which may lead to decreased glucose uptake and transport [66]. Similarly, in mice on a high-fat diet, it was found to lead to obesity and impaired glucose metabolism, accompanied by suppressed expression of *Gs3kβ* [67]. *Glut2*, an insulin target that controls glucose transport across the plasma membrane, showed decreased expression, which may affect insulin action and consequently lead to altered plasma glucose levels [68]. PM_2.5_ produces similar results to O_3_ exposure by disrupting the classical IRS-1/AKT signaling pathway in hepatocytes, causing the down-regulation of *Glut2* and *Glut4* expression and ultimately the disruption of glucose metabolism [69]. *G6pc* and *Fbp1*, key genes in hepatic gluconeogenesis, showed reduced expression, which may lead to decreased gluconeogenesis [69]. Wan et al. showed that low levels of lead exposure caused oxidative stress in the body, affecting *G6pc* and *Fbp1*, key enzymes in hepatic gluconeogenesis, leading to impaired FPG and hyperglycemia [70]. Furthermore, exposure to fine particle pollutants can also elevate the expression of *G6pc* in brain cells, resulting in gluconeogenesis metabolic disorder [71]. Additionally, *Pygl*, a critical enzyme that is involved in glycogen synthesis and glycogenolysis [72], showed reduced expression, which may cause an imbalance between glycogen synthesis and glycogenolysis, thereby affecting glucose utilization [73]. A study in male Swiss mice found that hepatic glucose and lipid homeostasis was impaired under conditions of exposure to chlorinated organic toxicants and *Pygl* expression was downregulated through activation of the AKT serine/threonine kinase 1 pathway [74]. Taken together, these findings suggest that decreased expression of these genes impairs hepatic glucose metabolism capacity, thereby affecting physiological processes in the body.

Most of the metabolic effects of fructose are due to its uncontrolled and rapid utilization by the liver and its direct catabolism by the action of fructokinase, which has a profound effect on carbohydrate and lipid metabolism [75]. Fructose in the liver generates fructose 1-phosphate through the action of fructokinase, which is then cleaved under the catalysis of aldolase B to glyceraldehyde and dihydroxyacetone phosphate, the latter of which enters the glycolytic pathway [76]. Glyceraldehyde, however, undergoes a series of transformations to form 3-Phosphoglyceric acid at the energy production stage of the glycolysis pathway [77]. In our study, we found that after O_3_ exposure, fructose content decreased and 3-Phosphoglyceric acid content increased in the livers of mice, which, according to the theoretical basis above, implies an increase in the catabolism of fructose by the liver. It is further used in the liver for glucose production, lipogenesis, glycogen synthesis, and energy supply [78]. Abnormal fructose metabolism is partly responsible for the increase in plasma glucose due to O_3_ exposure. For the differential metabolite methylmalonic acid, its abnormal metabolism disrupts normal glucose and glutamate metabolism in the mouse liver and may act by affecting the tricarboxylic acid cycle pathway [79].

As a strong oxidizing agent, O_3_ can induce the excessive production of intracellular ROS, such as superoxide and hydrogen peroxide, disrupting the redox balance [80]. Taking advantage of this characteristic, we used H_2_O_2_ to induce oxidative stress in HepG2 cells, mimicking the similar effects caused by O_3_ exposure. Research by Vašková et al. has revealed that O_3_ may induce endoplasmic reticulum stress through the PERK-eIF2α and IRE1-JNK pathways, promoting the unfolded protein response (UPR) and apoptosis [81]. Endoplasmic reticulum stress has been proven to be associated with pancreatic islet β-cell dysfunction and hepatocyte apoptosis [82]. Our cell experiments confirmed that after exposure, hepatocytes would produce excessive ROS and undergo apoptosis, further inducing insulin resistance, while pancreatic islet β cells showed abnormal glucose tolerance. Studies have indicated that regulators of apoptotic signaling events in hepatocytes can also modulate the insulin signaling pathway [83]. Moreover, liver apoptosis is a key event in the pathophysiology of diabetes, which can account for the adverse outcomes of insulin resistance and decreased glucose tolerance observed in both our in vivo and in vitro experiments following O_3_ exposure.

## 5. Conclusions

Our research demonstrates that long-term exposure to O_3_ causes an elevation in plasma glucose levels in adult female mice, increasing their risks of developing insulin resistance and glucose intolerance. This is because the oxidative stress generated in the body due to O_3_ exposure triggers excessive apoptosis of liver cells, thereby leading to disorders in hepatic glucose metabolism and abnormal expression of metabolite-related genes. This study provides a new target for the treatment and prevention of diabetes induced by O_3_ exposure. These tests can be used as criteria for diagnosing prediabetes and offer experimental support for evaluating the health risks associated with O_3_ exposure.

While our study provides novel insights into O_3_-induced metabolic dysfunction in females, it does not address potential sex-specific differences. Future studies should include male mice to determine whether O_3_’s diabetogenic effects are sex-dependent. We primarily investigated hepatic metabolic dysfunction (glycogen dysregulation and fructose metabolism). A deeper examination of pancreatic β-cell function (e.g., insulin secretion dynamics and islet morphology) could further clarify O_3_’s role in diabetes pathogenesis. Although we observed oxidative stress-mediated apoptosis and insulin resistance, the exact molecular pathways remain to be fully elucidated.

Future research can identify gender-specific biomarkers and therapeutic targets by comparing the responses of men and women to O_3_. Future research can test antioxidants (such as NAC and sulforaphane) or liver protectants to alleviate metabolic dysfunction caused by O_3_.

## Data Availability

The original data can be requested from the corresponding author (yuehuifeng@sxu.edu.cn) upon reasonable request, and a data usage agreement must be signed.

## Abbreviations

Abbreviation	Definition
AUC	Area under the curve
CHO	Cholesterol
CTD	Comparative Toxicogenomics Database
DAB	Diaminobenzidine
ELISA	Enzyme-linked immunosorbent assay
FINS	Fasting insulin
FPG	Fasting plasma glucose
Gapdh	Glyceraldehyde-3-phosphate dehydrogenase
HOMA	Homeostatic Model Assessment
H&E	Hematoxylin & Eosin
IHC	Immunohistochemical
LC-MS	Liquid Chromatography-Mass Spectrometry
LDH	Lactate dehydrogenase
LDL-C	Low-density lipoprotein cholesterol
OPLS-DA	Orthogonal Partial Least Squares Discriminant Analysis
qPCR	Quantitative real-time PCR
SI	Stimulation index
TG	Triglyceride
VIP	Variable important in projection

## Appendix A

### Appendix A.1. Exposure Concentration Basis

The exposure concentrations adopted are based on the following studies. Previous studies have reported that ozone levels of 0:16–0:20 ppm resulted in impaired lung function in exercising adults receiving a short-term exposure (Avol et al. 1984 [84]; Folinsbee et al. 1984 [85]); in sedentary laboratory rats, four to five times the ozone concentration of 0.54 ppm–1.0 ppm was required to induce a pulmonary inflammatory response, which is comparable to that induced in human subjects exercising under controlled acute exposure conditions (Hatch et al. 1994, 2013 [86,87]).

### Appendix A.2. Real-Time Quantitative PCR Experimental Method

Total RNA from the cortex was extracted using Trizol reagent (Promega, Madison, WI, USA) according to the manufacturer’s instructions. Total RNA was then treated with DNase I (Promega, Madison, WI, USA), and cDNA was synthesized using a First Strand cDNA Synthesis kit (Promega, Madison, WI, USA). The cDNA product was stored at −80 °C until used. qPCR was performed using Maxima SYBR Green qRT-PCR Master Mix kit (Promega, Madison, WI, USA) in a Real-Time PCR qTOWER 2.2 system (Analytik Jena AG, Jean, Germany), with *glyceraldehyde-3-phosphate dehydrogenase* (*Gapdh*) as the internal control. Each reaction mixture (20 µL) contained 0.5 µL of each primer, 2 µL of cDNA, 7 µL of RNase-free H_2_O, and 10 µL of SYBR Green (Promega, Madison, WI, USA).

### Appendix A.3. Gene Primers

**Table A1:** Gene primers.

Gene	Sequence (5′ → 3′)	
*Gs3kβ*	sense:	GGCTGTGTGTTGGCTGAATTGTTG
	antisense:	TTTGCTCCCTTGTTGGTGTTCCTAG
*Glut2*	sense:	ACAGTCACACCAGCATACACAACAC
	antisense:	CCGAGCCACCCACCAAAGAATG
*G6pc*	sense:	GACTGTGGGCATCAATCTCCTCTG
	antisense:	GCTGTTGCTGTAGTAGTCGGTGTC
*Fbp1*	sense:	GCTCTATGGTATCGCTGGCTCAAC
	antisense:	GACACAAGAACACAGGTAGCGTAGG
*Pygl*	sense:	GTCATTCCAGCCACAGACCTATCG
	antisense:	GCTTCCTCTGCCATCTCCACATTG
*Gys2*	sense:	GCCCTCCTCAGTACCACCTTCTC
	antisense:	CCTCTCAGCCTCCTCTTCCTCATC
*Agtr1a*	sense:	GGACACTGCCATGCCCATAACC
	antisense:	GTAGACAGGCTTGAGTGCGACTTG
*Pik3r1*	sense:	GGGAAGCGAGACGGCACTTTC
	antisense:	TCTACCACTACGGAGCAGGCATAG
*Insr*	sense:	ATCCGCCGCTCCTATGCTCTG
	antisense:	GAGTTGCCTCAGGTTCTGGTTGTC
*Egfr*	sense:	TCCTGATTGGTGCTGTGCGATTC
	antisense:	CTGGCAGTTCTCCTCTCCTCCTC
*Tnf*	sense:	CGCTCTTCTGTCTACTGAACTTCGG
	antisense:	GTGGTTTGTGAGTGTGAGGGTCTG
*Cd40*	sense:	TGTGATTTGTGCCAGCCAGGAAG
	antisense:	TTCTTAACCCGAAGCCCTTGATTGG
*Irs1*	sense:	GACGGGTTGCAGGAGGTGTTTG
	antisense:	TGGCAGGAGAGTGGTGGAGTTG
	antisense:	AGAAGAAGAGGCTGTGGAGGATGG
*Irs2*	sense:	TGTGATTTGTGCCAGCCAGGAAG
	antisense:	TTCTTAACCCGAAGCCCTTGATTGG

### Appendix A.4

**Table A2:** The genes related to ozone-related diseases retrieved by CTD.

Disease	Gene
Diabetes Mellitus	A|ADIPOQ|ADRB1|ALPK1|AOC3|CAT|CP|CPT1A|CYBB|FN1|IRS1|LEPR|MAP3K5|MT2A|NCF1|PON1|PPARG|PTGS2|RAC1|SOD1
Glucose Intolerance	AGT|AGTR1A|AHR|CD36|CNR1|CYP1B1|FRK|GPX4|IKBKB|INSR|LEP|LEPR|METRNL|NFE2L2|NONO|NR5A2|PIK3R1|PRDX4|PRKAA1|RAPGEF3|SCD1|SQSTM1|TLR4|XBP1
Insulin Resistance	ACACB|ADIPOQ|ADM|ADRB2|AHR|AR|C3|CASP1|CCL2|CD163|CD36|CD40|CD68|CPE|EGFR|GNAS|GPX3|HMGA1|HMOX1|HSD11B1|IGFALS|IGFBP2|IKBKB|INS1|INSR|IRS1|IRS2|ITGAM|LEP|LEPR|LIPC|MC4R|METRNL|NFE2L2|NLRP3|NOS3|NR4A1|NR4A3|PIK3R1|PLTP|PPARA|PPARG|PRKAA1|PRKAR1A|PTPN1|RETN|SCD1|SELENOP|SLC2A4|SOD2|SQSTM1|SREBF1|SREBF2|TNF|XBP1|ZC3H10
Hyperglycemia	A|ADIPOQ|ADM|AGER|CCL2|CD163|CD40|CNR2|COL3A1|FBN1|FCGR3B|GCK|GPX1|HMGA1|HNF1A|HSD11B1|IL6|INS1|INSR|IRS2|ITGAM|LEP|LEPR|NFE2L2|NOS3|NQO1|PRDX4|PRKAR1A|PRKCB|PTGS2|SP1|TERT|ZC3H10
Diabetes Mellitus, Type 2	A|ADAMTS9|ADCY5|ADIPOQ|AKT1|AKT2|ATF3|ATP2A2|ATP2A3|AUTS2|BAX|BCL2|BCL2L1|BCL2L11|BRAF|C2CD4B|C3|CAPN10|CASP3|CASP8|CAT|CBS|CCND2|CD36|CDKAL1|CDO1|CMIP|CPT1A|CYP1A2|ECE1|EDN1|EDNRA|EDNRB|EGFR|ENPP1|ETS1|FAS|FTO|GCK|GCLC|GCLM|GLP1R|GP2|GPD2|GPX1|GSTM1|HBA1|HHEX|HK1|HMG20A|HMGA1|HMOX1|HNF1A|HNF1B|HNF4A|HP|HPX|ICAM1|ID1|IL13RA1|IL6|INPPL1|INS1|IRS1|IRS2|ITGA1|JADE2|KCNQ1|LEP|LEPR|LIPC|MAEA|MAT1A|MIR130B|MIR144|MIR17HG|MIR222|MIR409|MIR744|NFKB1|NOS2|NOS3|NOTCH2|OGG1|PAM|PEPD|PPARA|PPARG|PPARGC1A|PRKCB|PTPN1|RETN|S100A6|SCTR|SLC22A3|SLC2A1|SLC2A4|SMAD5|SNAP25|SOD1|SOD2|TCF7L2|TIMP1|TMEM18|TNF|TNFRSF1A|TNFRSF1B|UBE2E2|WFS1|ZC3HC1|ZFAND3

### Appendix A.5

**Table A3:** Differential metabolite screening.

Differential Metabolites	Mean Value of Control Group (ng/mL)	Mean Value of 1.0 ppm Group (ng/mL)	Fold Change	VIP
3-Phosphoglyceric acid	1.999034	3.052333	1.526904	1.262554
Fructose	144.1563	88.70745	0.615356	1.651555
Methylmalonic acid	2.684819	1.786713	0.665487	1.033505
